# Early- and later-phases satellite cell responses and myonuclear content with resistance training in young men

**DOI:** 10.1371/journal.pone.0191039

**Published:** 2018-01-11

**Authors:** Felipe Damas, Cleiton A. Libardi, Carlos Ugrinowitsch, Felipe C. Vechin, Manoel E. Lixandrão, Tim Snijders, Joshua P. Nederveen, Aline V. Bacurau, Patricia Brum, Valmor Tricoli, Hamilton Roschel, Gianni Parise, Stuart M. Phillips

**Affiliations:** 1 School of Physical Education and Sport, University of São Paulo, São Paulo, São Paulo, Brazil; 2 Department of Physical Education, Federal University of São Carlos, São Carlos, São Paulo, Brazil; 3 Department of Human Movement Sciences, Maastricht University, Maastricht, Limburg, Netherlands; 4 Department of Kinesiology, McMaster University, Hamilton, Ontario, Canada; West Virginia University School of Medicine, UNITED STATES

## Abstract

Satellite cells (SC) are associated with skeletal muscle remodelling after muscle damage and/or extensive hypertrophy resulting from resistance training (RT). We recently reported that early increases in muscle protein synthesis (MPS) during RT appear to be directed toward muscle damage repair, but MPS contributes to hypertrophy with progressive muscle damage attenuation. However, modulations in acute-chronic SC content with RT during the initial (1^st^-wk: high damage), early (3^rd^-wk: attenuated damage), and later (10^th^-wk: no damage) stages is not well characterized. Ten young men (27 ± 1 y, 23.6 ± 1.0 kg·m^-2^) underwent 10-wks of RT and muscle biopsies (vastus-lateralis) were taken before (Pre) and post (48h) the 1^st^ (T1), 5^th^ (T2) and final (T3) RT sessions to evaluate fibre type specific SC content, cross-sectional area (fCSA) and myonuclear number by immunohistochemistry. We observed RT-induced hypertrophy after 10-wks of RT (fCSA increased ~16% in type II, *P* < 0.04; ~8% in type I [ns]). SC content increased 48h post-exercise at T1 (~69% in type I [*P* = 0.014]; ~42% in type II [ns]), and this increase was sustained throughout RT (pre T2: ~65%, ~92%; pre T3: ~30% [ns], ~87%, for the increase in type I and II, respectively, vs. pre T1 [*P* < 0.05]). Increased SC content was not coupled with changes in myonuclear number. SC have a more pronounced role in muscle repair during the initial phase of RT than muscle hypertrophy resulted from 10-wks RT in young men. Chronic elevated SC pool size with RT is important providing proper environment for future stresses or larger fCSA increases.

## Introduction

Skeletal muscle mass is determined by the balance between protein turnover rates (muscle protein synthesis [MPS] and breakdown). Protein turnover is regulated to some degree by gene transcription from pre-existing post-mitotic myonuclei within fibres, which are thought to control a finite volume of cytoplasm (referred to as myonuclear domain or DNA unit [1]). However, myonuclear number can be increased through donation from muscle stem cells, also known as satellite cells (SC). SC undergo proliferation, differentiation, and fusion to muscle fibres under conditions that require increased transcriptional capacity [2, 3], such as muscle damage [4–6] and large increases in muscle fibre size [7–9].

We recently showed that the initial sessions of resistance training (RT) result in significant muscle damage requiring a post-resistance exercise (RE) MPS increase that appears directed toward repair [10]. However, muscle damage is progressively attenuated as RT progresses and MPS is, we proposed, directed more towards promoting muscle hypertrophy [10]. As SC are involved in repairing muscle damage and supporting hypertrophy, it is of interest to determine the response of these cells throughout a RT program. Previously, studies demonstrated that muscle damage-inducing RE protocols increased the number of SC 24-72h post-RE [5, 11]; while ablation of SC impairs regeneration of muscle tissue after injury [4] or exercise [12]. However, it is unclear whether the acute damage-induced expansion of SC translates into an increase in myonuclear content. Studies have reported that RE-induced muscle damage promote increases in SC pool size, but do not result in an increase in myonuclear content up to 120h post-RE bout [5] or even later-on (27 days post-RE bout) [13]. Thus, we propose that in a regular RT program, where muscle damage is mild-to-moderate after the first RE session but quickly and progressively decrease in magnitude through RT [10], the contribution of nuclei may be required only to support continuous muscle fibre growth (i.e., large magnitude of muscle fibre hypertrophy). A working theory is that as RT progresses and fibre size expands beyond the transcriptional capacity of existing myonuclei, SC nuclei may be required [7–9, 14, 15].

In the current study we aimed to extent upon previous results [10] by examining the changes in type I and type II muscle fibres SC content before and after RE sessions in different phases of a RT program: at the start (week 1, T1) where skeletal muscle repair is a dominant function of MPS; at an early phase (week 3, T2) where damage is attenuated compared to T1; and at a later phase (week 10, T3) where muscle damage is greatly attenuated. Additionally, we determined muscle fibre cross-sectional area (fCSA), myonuclear content and domain size in type I and II muscle fibres, at the same time points. We hypothesized that in the untrained state SC content would increase in response to damage with no detectable change in the number of myonuclei at an early time-point (i.e., third week, T2). However, as RT progresses and individuals experience progressively less muscle damage, we expected that fCSA will increase, SC pool expansion will be kept elevated chronically and increases in myonuclear number per fibre will be observed, but with no change in myonuclear domain size.

## Methods

### Participants

Muscle biopsies examined herein for immunohistochemical analysis were collected from 10 healthy young men who had previous experience in lower limb RT (but had not engaged in lower limb RT for at least 6 months prior to the beginning of the study) and were participants in a previous study [16]. The present study was approved by The Human Research Ethics Committee of the local University (608.754, March 27, 2014) and all of the procedures performed herein were in accordance with the Declaration of Helsinki. All subjects signed a written informed consent form before enrollment in the study.

### Experimental design

Participants performed lower body RT for 10-wks (twice a week, totaling 19 workouts). Muscle biopsies were obtained before (Pre) and post-RE (48h) at three training-phases throughout the experimental period: first workout (T1), fifth workout (T2) and nineteenth workout (T3). Pre biopsies at T2 and T3 were taken 120h after the last training bout. Muscle biopsies were used for immunohistochemical analyses specific per fibre type (I and II) including fCSA, myonuclear content and domain size, and SC content. As the present investigation aimed at extending the results of our previous study, it should be noted that our first RE session (at T1) promoted the highest magnitude of muscle damage (indicate by Z-band streaming, and indirect markers, such as loss in muscle strength, increase in muscle soreness, and muscle proteins in the blood), and even though our protocol was always maximum, muscle damage was attenuated at T2 and insignificant at T3 [10].

### Diet and physical activities control

The diet was standardized on the days of biopsies (22% protein, 41% carbohydrates, and 37% lipids). Additionally, each participant ingested 25g of isolated whey-protein immediately after every RT bout (throughout the RT period). During the experimental period, participants were instructed to maintain their normal eating habits, to not consume any other supplements, and to refrain from other physical activities 72h before T1 and throughout the study period.

### Resistance training

The RT protocol consisted of 3 sets of 45° leg-press followed by 3 sets of leg extension exercises with 90s rest between sets and exercises. Each set consisted of 9–12 maximum repetitions (performed to volitional fatigue), inducing constant load adjustments through sets and RE sessions to maintain the maximum repetition range [10, 16]. Therefore, at evaluations weeks (i.e., T1, T2, and T3) the RE was performed at the same relative (maximum) load.

### Muscle biopsy

Muscle samples were collected from the vastus lateralis (VL) using the percutaneous biopsy technique with suction, performed by an experienced medical doctor. Sequential samples were performed on alternating legs; however, Pre biopsies were always performed in the same leg at T1, T2, and T3. This procedure was conducted so the same sequence of legs were biopsied at T1, T2 and T3 and thus, reducing variability among testing weeks. The biopsy procedure involved administration of local anesthesia [2–3 ml of 1% Xylocaine (lignocaine)] and via small incision, ~20–30 mg of muscle was removed using a Bergström needle. The tissue was dissected free from blood and connective tissue and placed in optimum cutting temperature (OCT) embedding medium with its fibres perpendicular to the horizontal surface and quick-frozen in isopentane cooled by liquid nitrogen. Immediately after preparation, all tissue were frozen in liquid nitrogen and stored at −80°C until analysis.

### Immunohistochemical analyses

Muscle cross-sections (7 μm) were prepared from samples mounted with OCT, and then brought to room temperature and fixed in 2% paraformaldehyde (PFA) for 10 min, washed and blocked then for 90 min in PBS (containing 2% bovine serum albumin [BSA], 5% fetal bovine serum, 0.02% Triton X-100, 0.1% sodium azide and 5% goat serum [GS]). For quantification of type I and type II SC content, the slides were incubated with primary antibodies against Pax7, laminin, MHCI and MHCII. All secondary antibodies were from Invitrogen (Burlington, Canada). Detailed antibody information is found in Table 1.

**Table 1:** Antibodies information.

Antibody	Species	Source	Clone	Primary	Secondary
**Anti-Pax7**	Mouse	DSHB	Pax7	Neat	Alexa Fluor 594, goat anti-mouse, 1:500
**Anti- laminin**	Rabbit	Abcam	ab11575	1:500	Alexa Fluor 488, goat anti-rabbit, 1:500
**Anti-MHCI**	Mouse	DSHB	A4.951 Slow isoform	Neat	Alexa Fluor 488 goat anti-mouse, 1:500
**Anti-MHCII**	Rabbit	Abcam	ab91506 Fast isoform	1:1000	Alexa Fluor 647 goat anti-rabbit, 1:500

Detailed information on primary and secondary antibodies used for immunofluorescence of muscle cross sections.

**Fig 1:**
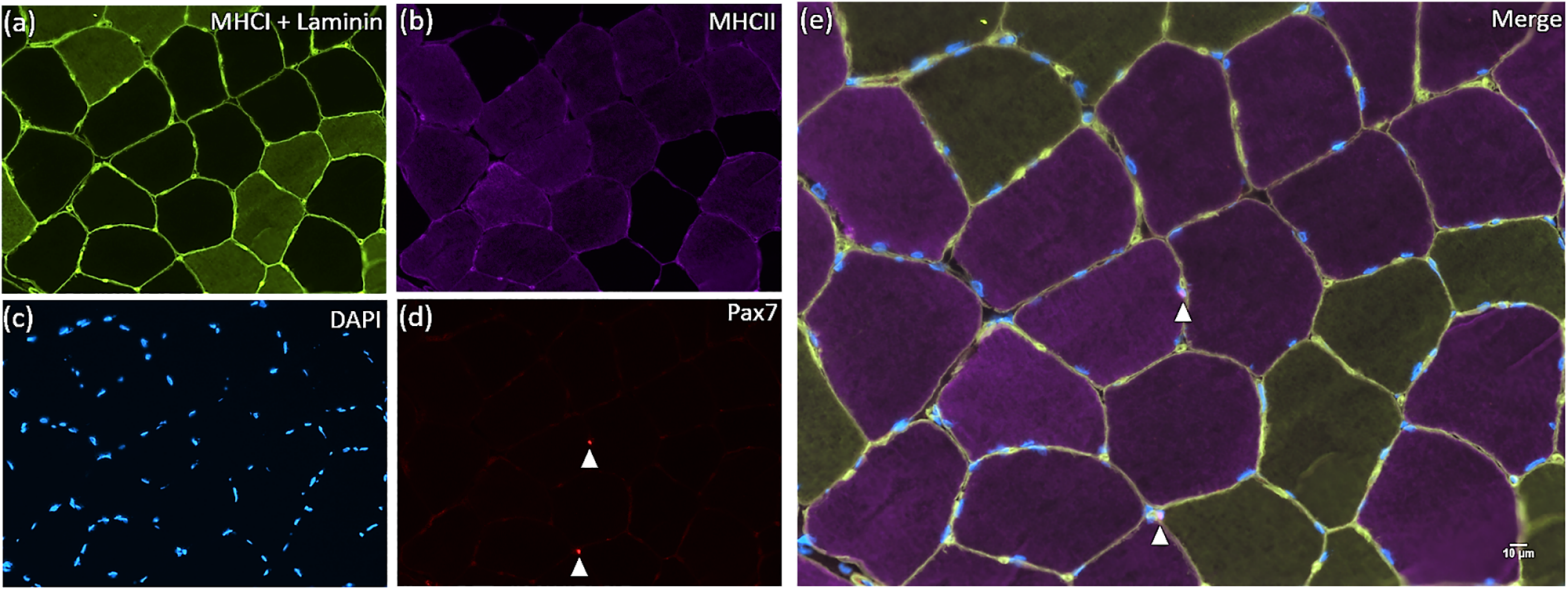
Representative image of the immunofluorescent stain. (a) MHCI/Laminin; (b) MHCII; (c) DAPI; (d) Pax7; and (e) MHCI/Laminin/MHCII/Pax7 (Merge). Arrows denotes SC (upper arrow: SC cell associated with a type II fibre; lower arrow: SC cell associated with a type I fibre).

Nuclei were visualized with 40.6 diamidino-2-phenylindole (DAPI 1:20 000, Sigma-Aldrich, Oakville, ON, Canada) before coverslipping slides with fluorescent mounting media (Dako, Burlington, ON, Canada). The images were observed with a Nikon Eclipse 90i microscope with a magnification of 20X and captured with a fluorescent fresh Photometrics camera SNAP HQ2 (Nikon Instrument, Melville, New York, USA). The analyses were completed using the software Nikon NIS-Elements AR software (Nikon Instruments, New York, USA) on a large image scale. A representative image is depicted in Fig 1. For fibre type specific fCSA measurements, the area of each individual muscle fibre was calculated using computerized planimetry (i.e., the perimeter of each individual fibre delimited by fluorescent laminin was manually contoured using a high dpi mouse). All areas selected for analyses were free of freeze fracture artifact and care was taken that the longitudinal fibres were not considered in the analysis. Muscle fibres in the periphery of the muscle cross-sections were also excluded from the analysis. The number of myonuclei in type I and II muscle fibres was counted and normalized by the respective number of muscle fibres analysed. Myonuclear domain size was determined as a ratio between fibre type specific fCSA and the normalized number of myonuclei per fibre type. The number of SC associated with type I and II muscle fibres was determined via the co-localization of Pax7^+^ and DAPI in each muscle fiber type. Afterwards, we normalized the fibre type specific SC content per number of type I and II muscle fibres analysed. An average (range) of 49 (35–50) type I fibres and 50 (50–50) type II fibres were analyzed for fCSA, myonuclear content and domain size. For fibre type specific SC content, an average (range) of 99 (48–201) type I fibres and 235 (63–552) type II fibres were analysed. All immunofluorescence analyzes were completed by the same experienced investigator in a blinded manner. Reliability (typical error) between measurements was good (fCSA: 94.3 *μ*m^2^; myonuclear content: 0.15 myonuclei/fibre; and SC content: 0.01 SC/fibre).

### Statistics

Analyses of fCSA, myonuclear content and myonuclear domain were performed using mixed model analysis having training-phase (T1, T2, and T3) as fixed factor and subjects as a random factor. For SC analyses, we used a mixed model assuming acute time changes (Pre and 48h) and training-phase (T1, T2 and T3) as fixed factors and subjects as a random factor. When a significant *F* value was found, a Fisher’s LSD post-hoc was applied for pair-wise comparisons. Pearson’s product moment correlation was used for association between variables also including data from Damas et al. [10]. Correlation coefficients |*r*| < 0.2 were considered small; 0.2 < |*r*| < 0.7, moderate; and |*r*| > 0.7, high. *P* was set as ≤ 0.05. Results are presented as mean ± SD. Figs are presented as box and whisker plots including the median (line), mean (cross), inter-quartile range (box), and 95% CI (tails).

## Results

### Fibre cross-sectional area, myonuclear content and domain size

RT resulted in a significant increase in type II muscle fCSA (T3 vs. T1, ~16%, *P* = 0.025; and T3 vs. T2, ~14%, *P* = 0.035) and a non-significant increase in type I muscle fCSA (~8%, *P* > 0.05, Table 2). There was no significant change in myonuclear content or domain size for either type I or II muscle fibres throughout 10-wks of RT (*P* > 0.05; Table 2).

**Table 2:** Type I and II muscle fibres cross-sectional area (fCSA), myonuclear number and domain size at the first (T1), third (T2) and tenth (T3) weeks of resistance training.

	Fibres	T1	T2	T3
**fCSA per fibre (μm**^**2**^**)**	Type I	4175 ± 553	4024 ± 727	4424 ± 582
	Type II	4643 ± 390	4537 ± 789	5304 ± 695 *
**Myonuclear number per fibre**	Type I	2.9 ± 0.4	3.1 ± 0.7	2.7 ± 0.4
	Type II	3.2 ± 0.5	3.3 ± 0.8	3.2 ± 0.8
**Myonuclear domain size per fibre (μm**^**2**^**)**	Type I	1505 ± 184	1356 ± 393	1578 ± 265
	Type II	1476 ± 350	1391 ± 373	1621 ± 318

Values are expressed as mean ± standard deviation.

*Significantly different from T1 (*P* = 0.025) and T2 (*P* = 0.035).

### Satellite cell content

**Fig 2:**
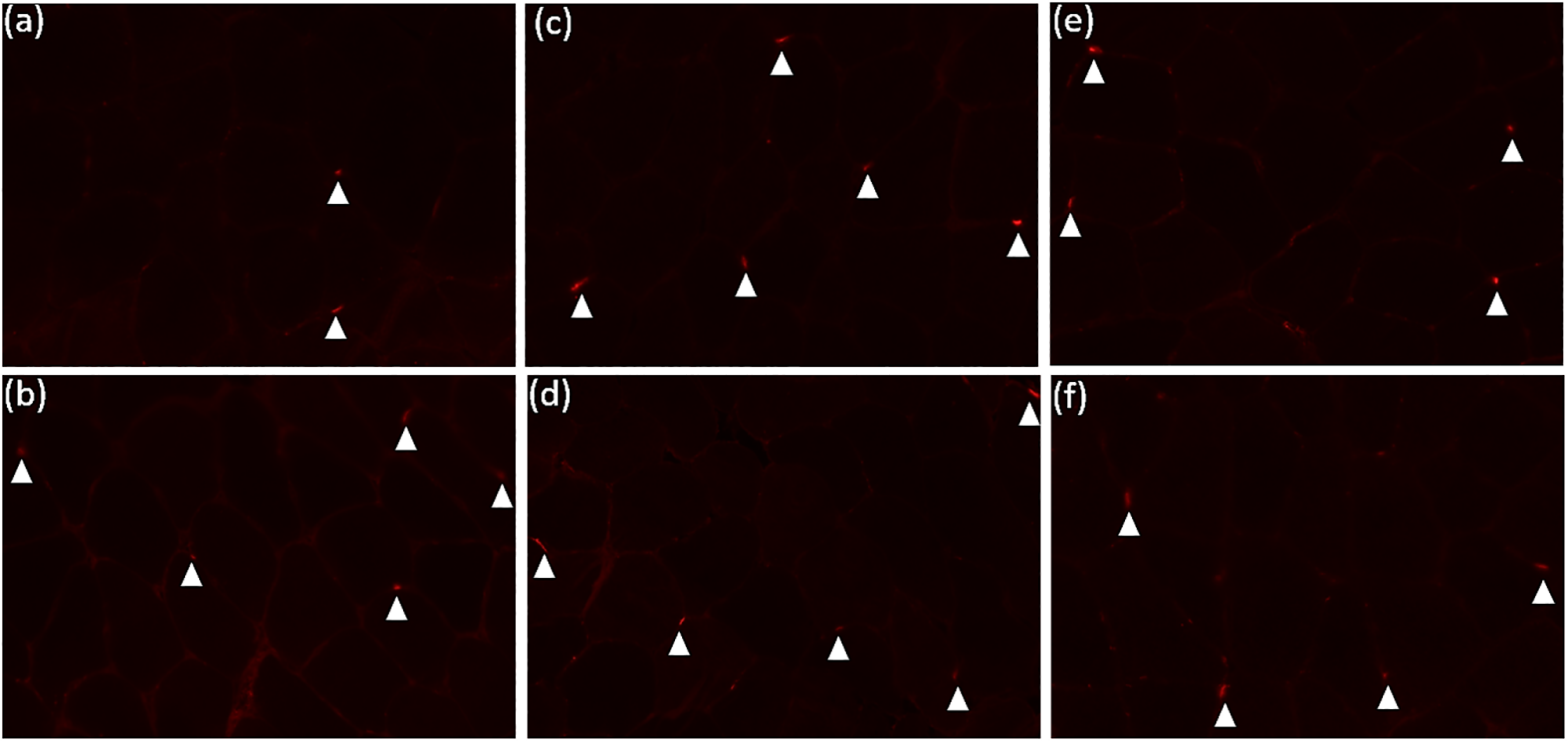
Pax7 stainings pre and 48h-post first (T1), fifth (T2) and nineteenth (T3) workouts. (a) Pre T1; (b) 48h T1; (c) Pre T2; (d) 48h T2; (e) Pre T3; (f) 48h T3. Arrows denotes SC.

An image depicting Pax7 stainings in all conditions analysed herein (i.e., pre and 48h at T1, T2 and T3) is showed in Fig 2.

**Fig 3:**
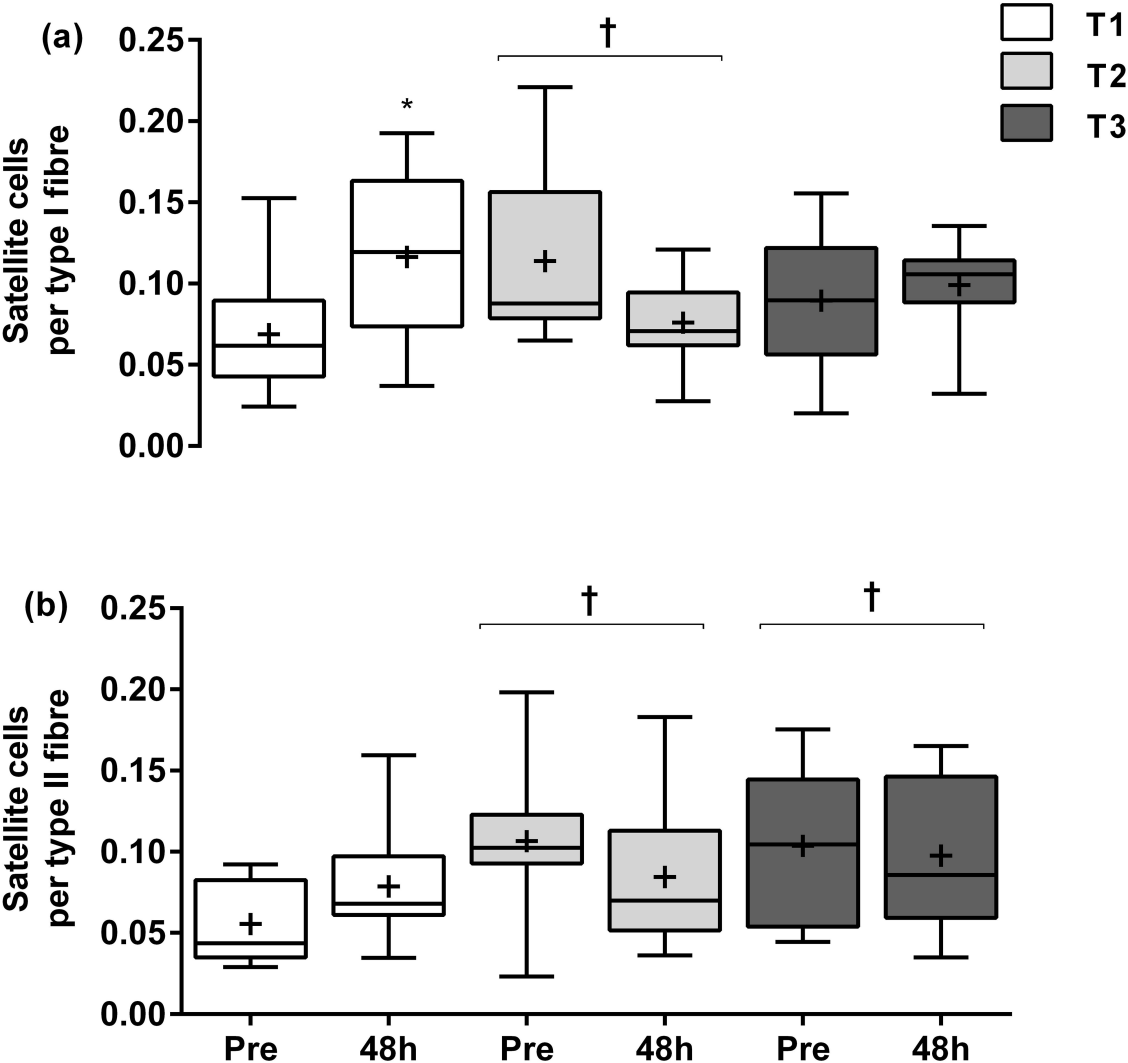
Satellite cells per type I (a) and type II (b) muscle fibres at rest (Pre) and after (48h) following a single bout of resistance exercise at the first week (T1), third week (T2) and tenth week (T3) of resistance training. * Significantly different from Pre at T1 (*P* = 0.014). † Significantly different from T1 (*P* < 0.05). Values are presented as median (line) with inter-quartile range (box), maximum and minimum values, and mean (+).

The number of SC per type I muscle fibres increased significantly 48h post-RE (0.116 ± 0.051) when compared with Pre values (0.069 ± 0.037, ~69%, *P* = 0.014) at T1. However, there were no further significant acute (pre to 48h post) changes in type I muscle fibre SC content at T2 and T3 (T2: 0.114 ± 0.054; T3: 0.089 ± 0.044) to 48h (T2: 0.076 ± 0.026, *P* > 0.05; T3: 0.099 ± 0.028, *P* > 0.05). Regarding the differences among training-phases, the number of SC in type I muscle fibres was significantly different than at T1 (~65%, *P* < 0.04), with no further change at T3 (~30%, *P* > 0.05) (Fig 3A). For SC per type II muscle fibres, there were no significant changes in SC from Pre (0.056 ± 0.024) to 48h at T1 (0.079 ± 0.037, *P* > 0.05), T2 (Pre: 0.107 ± 0.048; and 48h: 0.085 ± 0.044, *P* > 0.05) or T3 (Pre: 0.104 ± 0.047; and 48h: 0.098 ± 0.046, *P* > 0.05). However, when comparing training-phases, we observed a significantly greater number of SC in the type II muscle fibres at T2 and T3 than T1 (main training-phase effect, ~92%, *P* = 0.048 and ~87%, P = 0.017, respectively) (Fig 3B).

### Correlation analysis

Table 3 depicts variables that resulted in a significant association at some point during RT. Specifically, significant correlations were found between integrated MPS and type I and II muscle fibres myonuclear number at T3 (*r* = 0.730, *P* = 0.040 and *r* = 0.887, *P* = 0.001, respectively).

**Table 3:** Correlations between myonuclear number in type I and II muscle fibres vs. MPS increase (0-48h) post resistance training bout in T1, T2, and T3.

Variable		Integrated MPS T1 (%·day^-1^)	Integrated MPS T2 (%·day^-1^)	Integrated MPS T3 (%·day^-1^)
**Type I fibres myonuclei at T1**	*r*	-0.454		
	*P*	0.219		
**Type I fibres myonuclei at T2**	*r*		0.286	
	*P*		0.493	
**Type I fibres myonuclei at T3**	*r*			**0.730**
	*P*			0.040*
**Type II fibres myonuclei at T1**	*r*	-0.025		
	*P*	0.944		
**Type II fibres myonuclei at T2**	*r*		0.468	
	*P*		0.243	
**Type II fibres myonuclei at T3**	*r*			**0.887**
	*P*			0.001*

Integrated MPS: myofibrillar protein synthesis considering the first 48h (i.e., 0 to 48h) after a single resistance exercise bout; T1: first week, T2: third week, T3: last week of training. MPS data from Damas et al. [10].

*Significant (*P* < 0.05) correlation. Bold numbers indicate high correlation between variables.

## Discussion

The present study analysed acute and chronic changes in the SC pool in relation to different phases of a RT program (within-subjects design), each one of them (i.e., T1, T2 and T3) with distinct magnitudes of muscle damage and MPS orientation (repair- or hypertrophy-oriented), as we previously reported [10]. In the untrained state (i.e., T1), SC content increased pre to 48h post-RE, but no change was detectable in myonuclear content at an early time point (i.e., pre T2). With RT progression, fCSA increased (T3), although not to an extent that appeared to require the addition of new myonuclei to both fibre types; however, the initial SC pool expansion remained at this level chronically.

According to the myonuclear domain theory, early stages of skeletal muscle fibre hypertrophy are supported by pre-existing myonuclei, however, during extensive muscle fibre growth SC are required to supply additional myonuclei [7–9]. In the present study, we observed a significant increase in fCSA in response to 10-wks of RT (~16% in type II and ~8% [ns] in type I muscle fibres). However, the degree of muscle fibre hypertrophy observed was, most likely, insufficient to induce an appreciable increase in myonuclear content that could be detected by immunohistochemistry (Table 2). This is in agreement with previous studies employing similar RT duration, observing similar muscle fibre hypertrophy response [17, 18]. Although participants had not performed any RE in the previous 6 months before study onset, they did had previous experience with RT. Hence, it is possible that participants included in the current study had an enhanced number of myonuclei per fibre from their previous training experience. Subjects in our study had at baseline 2.9 ± 0.4 and 3.2 ± 0.5 myonuclei per type I and II muscle fibres, respectively. Work from Petrella et al. [9, 19] showed that the number of myonuclear per fibre can reach up to ~3.0 for 20–35 year old men, and, ~3.1 for extreme-hypertrophy responders following RT. Previous human studies suggest that myonuclear number does not change during a period of detraining and/or atrophy [8, 20, 21]. Therefore, it is plausible that our subjects did not require additional nuclei (i.e., pre-existing myonuclei were enough) to support the magnitude of fibre hypertrophy reported herein. Actually, the fact that MPS increase post-RE occurs rapidly (in the first hours after RE [22, 23]), it has to be induced by pre-existing nuclei within the muscle fibre. Interestingly, we observed that type I and type II myonuclear content at T3 correlate strongly with MPS at T3 (*r* = 0.730, and *r* = 0.887 for type I and type II muscle fibres, respectively) (MPS data from Damas et al. [10]), but not at any other time point during the RT program (i.e., T1 or T2) (Table 3). Previously, we showed that MPS at T3 strongly correlated with mixed muscle fibre hypertrophy [10]. Therefore, at the beginning of RT the stress imposed by RE is elevated, resulting in a high overall non-specific muscle protein turnover, which may explain the lack of significant correlation between myonuclei and the increase in myofibrillar protein synthetic response. With muscle adaptation throughout RT, stress/damage of each RE bout progressively decrease, and myonuclei seem to be more related to the increase in hypertrophy-oriented MPS. Thus, we suggest: 1) myonuclei seem to be ‘specialized’ with RT in promoting myofibrillar protein translation; and 2) the higher the number of myonuclei at the end of the 10-wks RT period, the higher was the increase in MPS response following the single bout of RE at T3 (which was correlated with mixed muscle fibre hypertrophy in our previous study [10]). Overall, pre-existing myonuclei were sufficient to provide the transcriptional activity necessary to support muscle fibre growth, especially with mitigation of muscle stress/damage and demanding for repair. If RT was extended beyond 10-wks, maybe fCSA were increased further requiring the addition of new nuclei donated by SC.

In the present study, SC number increased acutely (i.e., 48h post-RE) after the first RE session (~69% in type I and ~42% [ns] in type II muscle fibres). This is in line with previous studies showing similar acute expansion of the SC content 24 to 72h after an unaccustomed bout of isoinertial concentric-eccentric RE [11, 24]. However, the initial SC expansion did not promote detectable increases in myonuclear number during the first weeks of RT. It has been suggested that under normal minor damage-inflicting day-to-day activities, nuclear turnover is very low [25]. Therefore, in response to unaccustomed RE stress, it could be hypothesized that myonuclear turnover could have slightly increased at T1 (to support repair) requiring fusion of differentiated SC to muscle fibres to keep myonuclear content constant. Alternatively, a viable theory is that SC did not significantly fuse to muscle fibres, and the expansion on SC content initially in our non-severe damage-inducing RE protocol [10] could be related to extracellular matrix de-adhesion/remodelling [13, 26], secreting/producing factors within the extracellular matrix, such as matrix metalloproteinases [27] and collagens [28]. Future studies are warranted to further elucidate the precise role of SC pool size expansion during the initial stage of an exercise training program in humans.

In the current study we also examined the acute SC response early on during RT, i.e., after a period of adaptation (third week into RT; T2), where muscle damage is progressively being attenuated and MPS correlates with hypertrophy [10]. At this time point (T2), SC content did not increase acutely (pre to 48h) neither in type I nor II muscle fibres, what was also observed later-on RT (T3), when muscle damage was insignificant, and MPS highly correlated with muscle fibre hypertrophy [10]. These results appear to be in contrast with our previous publication showing acute (pre to 72h post-RE) SC pool size expansion following 16-wks of RT in healthy young men [29]. Despite the likelihood that there was no significant muscle damage from RE after 16-wks of RT, Nederveen et al. [29] did report a more extensive (~2-fold higher) muscle fibre hypertrophy (~34% increase in type II fCSA, and 13% in type I fCSA) in response to a longer RT period compared with the present study. Therefore, later RT-induced increases in SC content after an acute bout of RE [29] could be due to the demand for new nuclei to support extensive muscle fibre hypertrophy. In the present study, we show no acute (pre to 48h) changes in type I and/or type II muscle fibre SC content at T2 and T3, which is in line with data of attenuated muscle damage following RE at these time points [10] and the magnitude of muscle fibre hypertrophy observed during RT. Therefore, we propose that the early acute SC expansion is more related to the stress of exercise eliciting muscle damage from the unaccustomed bout performed at the onset of RT (muscle damage magnitude was the highest at T1 and progressively decreased [10]) than the magnitude of RE-induced muscle fibre hypertrophy depicted in the present study.

Chronically, our results show a maintenance of increased SC content in the early phase (third week) of RT program (pre T2 vs. pre T1: ~65%, ~92%, in SC content associated with type I and type II muscle fibres, respectively), and after 10-wks of RT (pre T3 vs. pre T1: ~30% [ns], ~87%, in SC content associated with type I and type II muscle fibres, respectively), which is in agreement with later stages of 10-12-wks training programs previously reported [17, 30]. Thus, with continuous RE stress throughout RT, SC content was maintained above basal levels, but no change in myonuclear content was evident. We suggest that SC pool size might become chronically elevated due to non-damage-inducing RE loading, which will be important for supporting possible future muscle repair/remodelling (or, maybe later-on RT, muscle hypertrophy), as previously suggested [12, 13, 31], or returning to quiescent state [32]. In addition, our results depict both the chronic maintenance of expanded SC content and the increase in fCSA to be more evident in type II muscle fibres, suggesting a possible role for SC in type II muscle fibre hypertrophy at further phases if we had extended the RT beyond 10-wks. Long-term maintenance of elevated type II fibre-associated SC pool size due to chronic exposure of muscle fibres to RT loading in young subjects has relevant implications on the importance of early-on in life engaging into RT practice, as elderly subjects not enrolled in regular RT have lower baseline SC content and show a blunted increase in type II fibre-associated SC content compared to younger counterparts after RE [11].

The present study has some limitations. Our relatively low power of analyses (*n* = 10) was possibly the reason why the ~42% increase in SC content in type II fibres pre to 48h post-RE at T1 was not statically significant (post-hoc analysis do show a *P* = 0.017 for this comparison, but no interaction effect was evident). We cannot comment on the activity of SC per se: activation, proliferation, and differentiation phases or even more specific cell cycle phases (e.g., G_0_/G_1_, S-phase, G_2_/M-phase) which could be analyzed through more specific stainings and/or flow cytometry methods. Further studies that include those analyses both acutely and chronically with RT would provide more insight on SC activity throughout RT. In addition, the results of the present study should be considered under the circumstances of 10-wks of RT in young men that had experience with RT in the past but were at least 6 months without performing RT. It should be noted, however, that several studies, e.g., [24, 29, 30] include ‘recreationally active’ participants; thus, possibly they are including participants with (at least) some previous experience in RT. Longer experimental designs, using different populations such as completely (their whole life) untrained individuals, elderly and frail subjects are also desired to investigate SC response and possible nuclei donation to muscle fibres.

In conclusion, results from the present study indicate that SC may have a more pronounced role in muscle repair during the initial phase of training than muscle fibre hypertrophy resulted from 10-wks of RT in young men with previous experience in RT. Furthermore, the chronic maintenance of elevated SC content due to repeated RE bouts is important providing proper environment to aid in future stresses or support larger muscle fibre hypertrophy following a more prolonged RT program.
