# Correction: Early- and later-phases satellite cell responses and myonuclear content with resistance training in young men

**DOI:** 10.1371/journal.pone.0193198

**Published:** 2018-02-14

**Authors:** The PLOS ONE staff

The Funding section is incorrect. The correct funding information is as follows: The present study was funded by the São Paulo Research Foundation (FAPESP) grants (#2013/21218-4 to CAL), CAPES-PROEX and Natural Science and Engineering Research Council (NSERC) of Canada (RGPIN-2015-04613 to SMP). SMP also acknowledges support from the Canada Research Chairs program. FD was supported by FAPESP grants (#2012/24499-1, #2014/19594-0, and #2016/24259-1). CU, HR and VT are supported by the National Council for Scientific and Technological Development (CNPq) grant (CU: #303085/2015-0 and #448387/2014-0; HR: #307023/2014-1; VT: #310823/2013-7). The funders had no role in study design, data collection and analysis, decision to publish, or preparation of the manuscript. The publisher apologizes for the error.
